# Detection and Measurement of the Intracellular Calcium Variation in Follicular Cells

**DOI:** 10.1155/2014/484656

**Published:** 2014-09-16

**Authors:** Ana M. Herrera-Navarro, Iván R. Terol-Villalobos, Hugo Jiménez-Hernández, Hayde Peregrina-Barreto, José-Joel Gonzalez-Barboza

**Affiliations:** ^1^Facultad de Informática, Universidad Autónoma de Querétaro, Campus Juriquilla, Avenida de las Ciencias s/n, 76230 Querétaro, QRO, Mexico; ^2^Centro de Investigación y Desarrollo Tecnológico en Electroquímica S.C., Pedro Escobedo, 76703 Querétaro, QRO, Mexico; ^3^Centro de Investigación e Ingeniería Industrial. Avenida Playa Pie de la Cuesta No. 702, Desarrollo San Pablo, 76703 Querétaro, QRO, Mexico; ^4^Departamento de Ciencias Computacionales, Instituto Nacional de Astrofísica, Óptica y Electrónica, Luis Enrique Erro No. 1, CP 72840, Tonantzintla, PUE, Mexico; ^5^Centro de Investigación y Tecnología Aplicada, Cerro Blanco No. 141, Colonia Colinas del Cimatario, 76090 Querétaro, QRO, Mexico

## Abstract

This work presents a new method for measuring the variation of intracellular calcium in follicular cells. The proposal consists in two stages: (i) the detection of the cell's nuclei and (ii) the analysis of the fluorescence variations. The first stage is performed via watershed modified transformation, where the process of labeling is controlled. The detection process uses the contours of the cells as descriptors, where they are enhanced with a morphological filter that homogenizes the luminance variation of the image. In the second stage, the fluorescence variations are modeled as an exponential decreasing function, where the fluorescence variations are highly correlated with the changes of intracellular free Ca^2+^. Additionally, it is introduced a new morphological called medium reconstruction process, which helps to enhance the data for the modeling process. This filter exploits the undermodeling and overmodeling properties of reconstruction operators, such that it preserves the structure of the original signal. Finally, an experimental process shows evidence of the capabilities of the proposal.

## 1. Introduction 

Calcium (Ca^2+^) is an ubiquitous intracellular ion signaling responsible for controlling many cellular processes [1, 2]. Ca^2+^ acts as second messenger triggering pathological events, such as cells injury and death, as well as participating in pathological conditions, such as hypertension, cardiac arrhythmia, hematological problems, muscular diseases, and hormonal disorders, among others [1, 3, 4]. The role of Ca^2+^ in these diseases has just begun to be understood. Some Ca^2+^ that induce pathological conditions have been found with the help of substances that interfere with the movement or activation of Ca^2+^ [3, 5]. Due to the importance of Ca^2+^, there are several numbers of optical and nonoptical techniques, which have been developed for analyzing Ca^2+^ either dynamics or concentration. Fluorescent microscopy techniques are frequently used to observe the variation of intercellular Ca^2+^ concentration applying chemical fluorescents indicator as markers. Those indicators stimulate the cells causing a fluorescence effect [6]. The fluorescence is detected by microscopy and CCD sensors. To compute the cells with the greatest fluorescence variations, the user must select them manually and analyze all the images in the sequence in order to determine the changes of fluorescence over time. However, it consumes time and human resources being susceptible to error measuring. By such circumstance, the process for segmenting the cells and the study of dynamics of intracellular Ca^2+^ represent the main objective in this paper.

An important step in the study of intracellular Ca^2+^ consists of the segmentation of each individual cell. The image segmentation results are difficult because the environmental changing conditions are usually uncontrollable. In the literature typically depending on the properties of the cells, the segmentation method is proposed. For instance, some works use neural networks approaches [7], hierarchical threshold [8], or multiscale morphology [9], just to mention a few.

Watershed-plus-marker approach, on the other hand, is the traditional image segmentation method based on morphological mathematical [10, 11]. The success of this method depends mainly on the correct detection of the image's markers. The markers can be detected manually or automatically. Automatic approaches help the specialist to save time and resources. However, there are factors that affect the performance of an automatic detection of markers such as noise, cells occlusions, and abrupt changes in the images. Those factors can lead these algorithms to over- and undersegmentation, that is, create regions containing partial or multiple cells. In this sense, several approaches have been developed for improving cell segmentation. In this study, we introduce a method to analyze automatically the intracellular calcium variation. This approach consists in two stages: (1) the image enhancement and cell segmentation and (2) the calcium variation modeling. The image enhancement is carried out with a top-hat filter, which homogenizes the luminance conditions. The process of cells segmentation is performed using the marked-controlled watershed transform and filters by reconstruction, which is used to detect markers efficiently, after the homotopy of the gradient image was computed. To measure the calcium intracellular variations, the volume of each marked cell is computed. The procedure consists of estimating the volume using the luminance intensities for each marked cell along the time. After, least squares fitting (LSF) method is applied to create a model of the behavior of the variation of the fluorescence. The behavior model created is considered as an exponential decreasing function. To enhance the development of the model of the behavior of calcium, a novel morphological filter named as medium filter is introduced. This filter smoothes the fluorescence measures, exploiting the under- and-overmodeling of reconstruction operator, preserving the information structure of original signal.

The paper is organized as follows. In the next section, a review of some morphological filters is presented. In Section 3, a method based on marked controlled watershed transform to detect automatically the cells is shown. In Section 4, we show the procedure to estimate the volume of each marked cell starting from the fluorescence intensity along time; after applied least squares fitting and morphological medium filter, the model of fluorescence behavior is created. Finally, results and conclusions can be found in the last section.

## 2. Concepts of Morphological Filtering

### 2.1. Basic Notions of Morphological Filtering

Mathematical morphology is mainly based on the so-called increasing transformations [12–14]. A transformation *T* is increasing if for all pairs of functions *f* and *g*, with *f* ≤ *g*⇒*T*(*f*) ≤ *T*(*g*). In other words, increasing transformations preserve the order of the relation. A second property is the idempotence; that is, a transformation *T* is idempotent if and only if *T*(*T*(*f*)) = *T*(*f*). The basic morphological filters are the morphological opening *γ*_*μB*_ and the morphological closing *φ*_*μB*_ with a given structuring element, where *B* represents the elementary structuring element (3 × 3 pixels, e.g.) containing its origin and *μ* is an homothetic parameter. Thus, the morphological opening and closing are given, respectively, by

(1) $$\begin{array}{c} \gamma_{\mu B}f\left(x\right)=\delta_{\mu B}\left(\varepsilon_{\mu B}\left(f\right)\right),\;\;\varphi_{\mu B}f\left(x\right)=\varepsilon_{\mu B}\left(\delta_{\mu B}\left(f\right)\right), \end{array}$$

where the morphological erosion *ε*_*μB*_(*f*(*x*))  and dilation  *δ*_*μB*_(*f*(*x*)) are expressed as *ε*_*μB*_ = *f* ⊖ *μB* : *x* ↦ inf⁡_*yϵμB*_ *f*(*x* + *y*) and *δ*_*μB*_(*f*) = *f* ⊕ *μB* : *x* ↦ sup⁡_*yϵμB*_ *f*(*x* − *y*).

Henceforth, the set *B* will be suppressed rendering, the expressions *γ*_*μ*_ and *γ*_*μB*_ are equivalent (*γ*_*μ*_ = *γ*_*μB*_). When the parameter *μ* is equal to one, all parameters are suppressed (*δ*_*B*_ = *δ*).

### 2.2. Opening (Closing) by Reconstruction

The notion of reconstruction is a very useful concept provided by MM. Reconstruction transformations are built by means of the geodesic transformations. In a gray-level case, the geodesic dilation *δ*_*f*_^1^(*g*) (resp., the geodesic erosion *ε*_*f*_^1^(*g*)) with *g* ≤ *f* (resp., *g* ≥ *f*) of size 1 given by *δ*_*f*_^1^(*g*) = *f*∧*δ*_*B*_(*g*) (resp., *ε*_*f*_^1^(*g*) = *f*∨*ε*_*B*_(*g*)) is iterated until idempotence. Consider two functions *f* and *g*, with *f* ≥ *g* (*f* ≤ *g*). Reconstruction transformations of the marker function *g* in *f*, using geodesic dilations and erosions, expressed by *R*(*f*, *g*) and R*(*f*, *g*), respectively, are defined by

(2) R(f,g)=lim⁡n→∞⁡εfn(g)=εf1εf1⋯εf1(g),R∗(f,g)=lim⁡n→∞⁡εfn(g)=εf1εf1⋯εf1(g).

When the marker function *g* is equal to the erosion or the dilation of the original function in (2), the opening and the closing by reconstruction are obtained:

(3) $$\begin{array}{c} {\tilde{\gamma }}_{\mu }\left(f\right)=\underset{n\to \infty }{\lim}\delta_{f}^{n}\left(\varepsilon_{\mu }\left(f\right)\right),\\ {\tilde{\varphi }}_{\mu }\left(f\right)=\underset{n\to \infty }{\lim}\varepsilon_{f}^{n}\left(\delta_{\mu }\left(f\right)\right). \end{array}$$

## 3. Automatic Detection of Cells

The watershed-plus-marker approach transformation is a traditional image segmentation method based in mathematical morphology [10, 13]. However, this transformation makes use of an extensive set of morphological filters. This transform is used for segmenting images avoiding the oversegmentation [11]. The oversegmentation criterion consists of setting an upper limit in the number of minima regions detected. This process is performed with the minima impositions over the markers, exploiting homotopy property of the operators. However, it needs to make some assumptions to use this approach. The most important assumption consists of the fact that that the minima represent the center of the object (in the cell case, the core of it); a second assumption consists of the gradient estimation, such that the fact that it could be performed with morphological operators.

### 3.1. Marker Detection

Due to the features of the images, the nucleus of each cell is used as regional minimum. As matter of fact, a regional minimum *M* of a gray-scale image *I* is a connected component of pixels with uniform altitude without lower neighbors. Before computing the minima, the nucleus of the cells is mainly dark, surrounded by brighter region composed by the cytoplasm. However, the local luminance conditions of each cell differ singly to each other, affecting the detection of the nucleus. For the homogenization of luminance conditions the top-hat transform is used as a local contrast correction filter.

**Figure 1:**
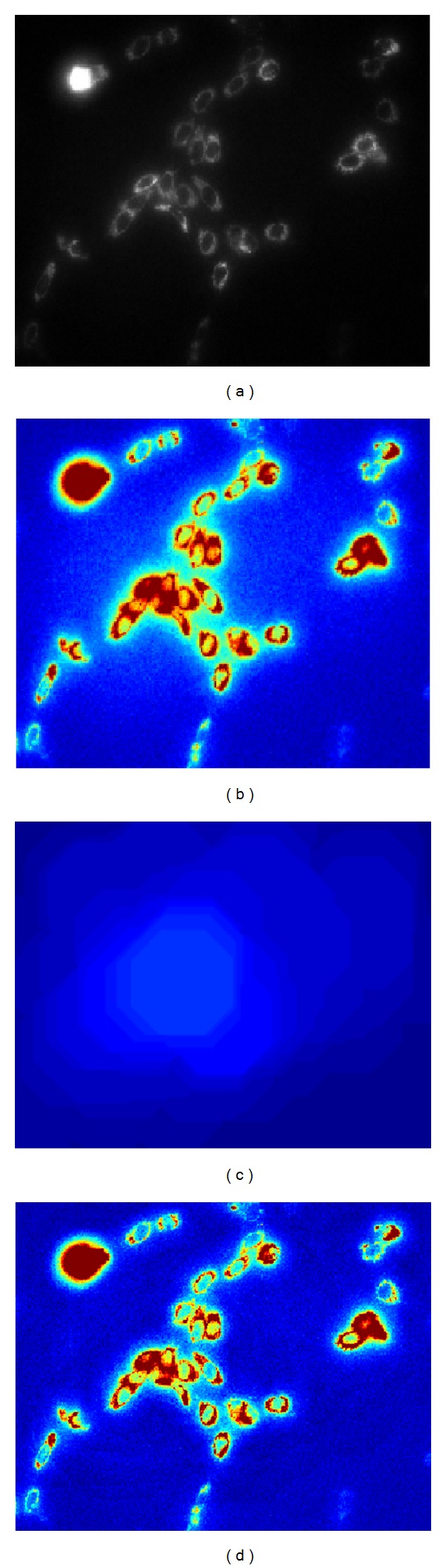
(a) Original image, (b) original image in pseudocolor before background correction, (c) opening morphological, and (d) image after background correction.

For the {*I*_*i*_}_*iiϵs*_ be sequence of image. The top-hat transformation is defined as follows:

(4) $$\begin{array}{c} {\text{Th}w}_{\lambda B}\left(I\right)=\left(I_{i}\right)\left(x\right)-\gamma_{\lambda }\left(I_{i}\right)\left(x\right), \end{array}$$

where the dimensions of structuring element are related with the luminance conditions of the scenario; in such a way, the luminance distribution in the image may be approximated with the morphological opening *γ*_*μ*_. Whenever the dimension of structuring element becomes proportionally similar to the image dimension, it would represent global luminance sources affectations; on the other hand, small dimensions represent local luminance variations effects. This process is illustrated in Figure 1, where each process step is showed and in certain steps the image has been coded in pseudocolor to point out the effects involved at each step.

**Figure 2:**
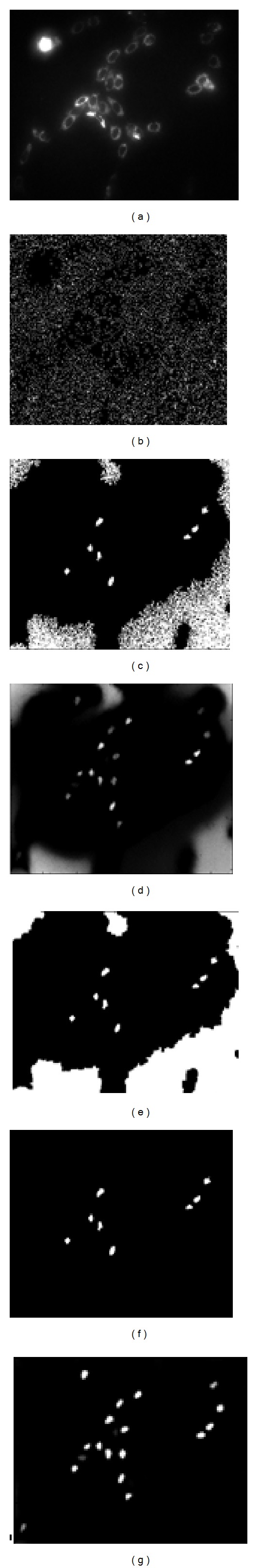
(a) Input image, (b) regional minima of original image, (c) minima obtained after applied closing by reconstruction, (d) function constructed from the minimum of the sequence of image, (e) morphological closing *φ*_*λ*=3_, (f) minima obtained by the difference: *M*_*i*_(*x*) = *M*_*i*_(*x*) − *γ*_*λ*=6_ *M*_*i*_(*x*), and (g) set of markers obtained by the function *I*_*m*_(*x*).

The direct appliance of the maxima transform detects all the maxima, including noise data, as it is appreciated in Figure 2(b). To avoid the extra minima detection, the image is enhanced with a closing by reconstruction operator. This operator allow grouping by all connected local minima, discarding the majority of noise effects. The closing by reconstruction uses a unitary structuring to approximate the original image. For illustration proposes in Figure 2(c) is appreciated the effect of apply the filter in the minima detection process. Observe that some cell nucleus are well detected, but others are omitted; due to the acquisition process the cytoplasm is not completely closed. This situation can be fixed using subsequent images, where additional minima are correctly detected. Thus, in order to obtain the maximum number of minima, a function that captures the occurrence of the minima in the sequence is constructed. Let {*I*_*i*_}_*i*∈*S*_ and {*M*_*i*_}_*i*∈*S*_ be the images of the sequence and the images containing the detected minima, respectively. *M*_*i*_(*x*) is a binary image such that it takes 1 value if the point *x* belongs to a regional minimum and 0 values otherwise. After using the subsequent frames, summation *I*_*m*_ is built as follows:

(5) $$\begin{array}{c} I_{m}\left(x\right)=\underset{i\epsilon S}{\sum }M_{i}\left(x\right). \end{array}$$

The surface *I*_*m*_ is drawn in Figure 2(d). Analyzing Figures 2(c) and 2(d), the majority of true minima are detected. Other big areas are pointed out in the figure. They are discarded by a thresholding criterion. In the case of study, each cell is typically about four pixel of radius, which can be discovered using an opening by reconstruction filter. As complementary, a closing operator with a structure of 3 pixels of dimension is used to connect insolated regions. The process is illustrated in Figure 2. First, a morphological closing of size 3 is applied to fill the small holes; the results can be appreciated in Figure 2(c) (before closing filter) and Figure 2(e) (after closing filter). Next, applying the summation *I*_*m*_ (Figure 2(d)), the minima are found, and finally the regions with big and small areas are discarded, denoting the cells.

### 3.2. Gradient Operator

The watershed-plus-marker approach makes use of the gradient operator to impose the markers. In this sense, the morphological gradient can be used as contrast detector. Let *I*(*x*) be a function defined in *Z* ^2^ and *B* the basic structuring element of 3 × 3 dimensions, centered at point *x*. Then, the transformation is defined as follows for a discrete space:

(6) $$\begin{array}{c} \nabla_{B}I\left(x\right)=\delta_{B}I\left(x\right)-\varepsilon_{B}I\left(x\right). \end{array}$$

There are other two versions of gradients in mathematical morphology, the internal and the external gradients defined, respectively, as follows:

(7) $$\begin{array}{c} \nabla_{B}I\left(x\right)=I\left(x\right)-\varepsilon_{B}I\left(x\right),\\ \nabla_{B}I\left(x\right)=\delta_{B}I\left(x\right)-I\left(x\right). \end{array}$$

**Figure 3:**
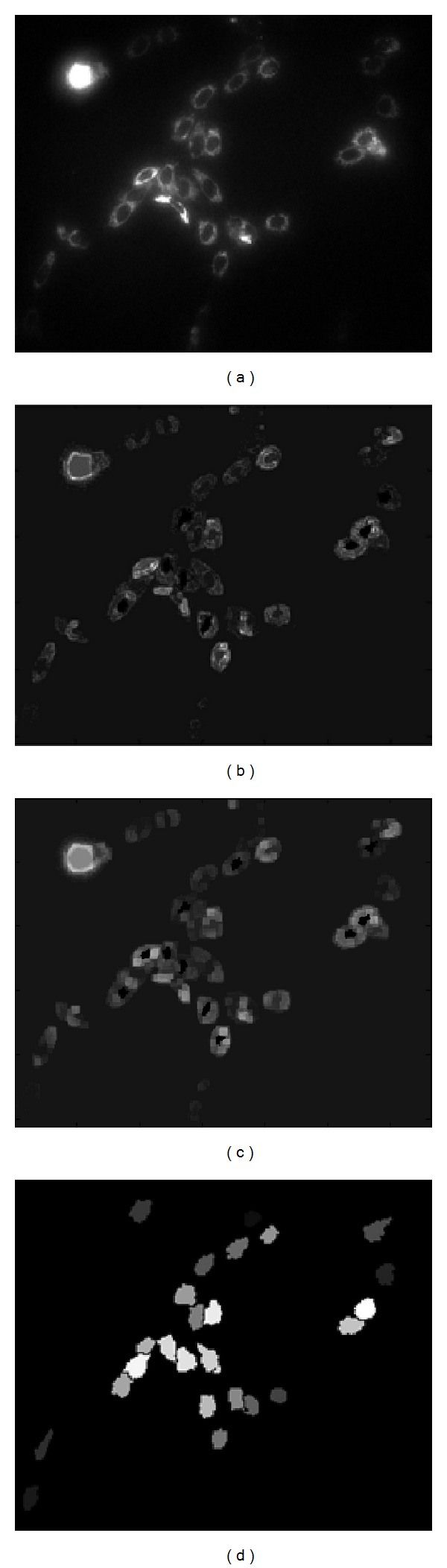
(a) Input image, (b) internal gradient, (c) external gradient, and (d) segmented cells.

Figures 3(b) and 3(c) show the internal and external gradients of the image in Figure 3(a). However, the indistinct use of any gradient approach has the consequence where the border should present double border. Typically they correspond to the cell generated among the nucleus of the cell and cytoplasm and the other one between the cytoplasm and the background of the image. The drawback to detect efficiently the true border in the image is an open task; then to deal with it, several tests have been applied to the images. Experimentally it is appreciated that external gradient offers smoother and thick borders (see Figure 3(c)), instead of internal gradient offering defined and clear borders as it is illustrated in Figure 3(c).

### 3.3. Imposed of Minima by Reconstruction

Once the markers cells signals are detected, these are imposed with their minima on the gradient image. To carry out this task the following procedure is performed. Let *M* and *g* be the set of markers computed as commented above and the gradient image, respectively. After, two news functions are built: the first one consists of a thresholding function *f*(*x*), which is defined as *f*(*x*) = 255 if *x* ∉ *M* and *f*(*x*) = 0 if *x* ∈ *M*, while the second one is built through the gradient image as *g*′(*x*) = *g*(*x*) if *x* ∉ *M* and *g*(*x*) = 0 if *x* ∈ *M*. Furthermore, the dual morphological reconstruction of *f*(*x*) inside of *g*′(*x*) is made, denoted by R*(*g*′, *f*). The function R*(*g*′, *f*) only has the minima of *M*, such that the watershed transformation is applied.  Figure 3(d) illustrates the results getting after applying watershed segmentation.

## 4. Modeling the Intracellular Calcium Dynamic

In this section, we deal with the problem of modeling the intracellular calcium dynamic. The procedure consists in three parts: the estimation of the calcium volume, the fitting of an exponential curve, and the calculus of the error.

### 4.1. Estimating Cell's Volume

The cell intensities are highly related to the amount of calcium contained in each cell. Then, the task of creating a model of the behavior of calcium in each cell is managed computing the volume of each cell in the image. The historical measures of time are used to represent the evolution of the dynamic of the variation of calcium for each particular cell. The historical measures of volumes are denoted by {*V*_*n*_(*i*)}_*i*∈*S*_, where the subindex *n* corresponds to a particular cell and *i* represent the particular volume for the time stamp *i*th. The volume is estimated with a discrete approximation of the integral as follows:

(8) $$\begin{array}{c} V=\overset{x_{f}}{\underset{x_{i}}{\int }}\overset{y_{f}}{\underset{y_{i}}{\int }}f\left(x,y\right)dy\;dx,\\ V\approx \overset{x_{f}}{\underset{x_{i}}{\sum }}\overset{y_{f}}{\underset{y_{i}}{\sum }}f\left(x,y\right)\Delta y\Delta x,\;\text{for}\;\;\Delta x=\Delta y=1. \end{array}$$

### 4.2. Modeling Intracellular Calcium Variations

**Figure 4:**
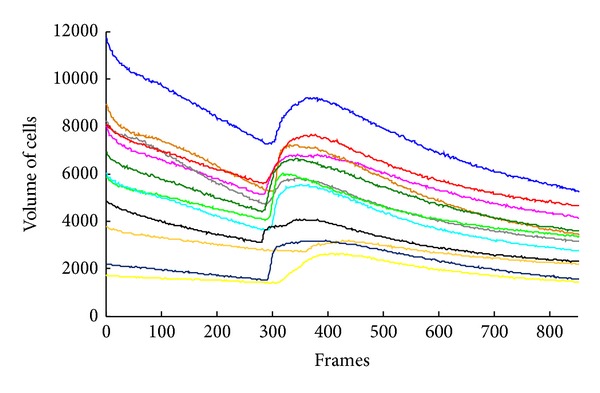
Cells volume curves over time showing an exponential decreasing behavior.

As it is appreciated in Figure 4, the dynamics of the calcium stimulus has an exponential behavior. Then, the purpose consists of creating a model of the decreasing behavior stimuli suffered by each cell, where the region of interest is located among the global maxima and the end of the signal. However, due to the noise, it is not possible to detect easily the maximum. To attenuate this inconvenience, an automatic process is performed detecting the maxima for the function {*V*_*n*_(*i*)}_*i*∈*S*_. The process consists of a sequential alternating filter in one-dimensional scenario. The alternating filter is constituted by a sequence of one closing by reconstruction followed by one opening by reconstruction ${\tilde{\varphi }}_{\mu L}\left({\tilde{\gamma }}_{\mu L}\left(V\right)\right)\left(i\right)$ where the size of *μ* is varied into the interval [0, *k*]. The filter undermodels the original signal, smoothing the signal wave and allowing the detection of the global maxima efficiently.

**Figure 5:**
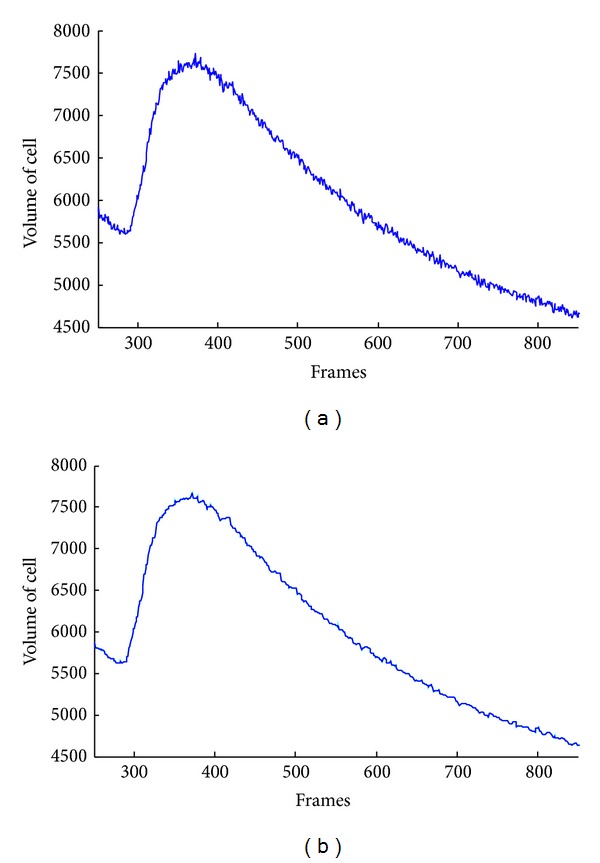
(a) Original signal that has multiple maxima caused by the noise interference. (b) Filtered signal presents a smoothing wave in which the global maximum is easy to detect.

**Figure 6:**
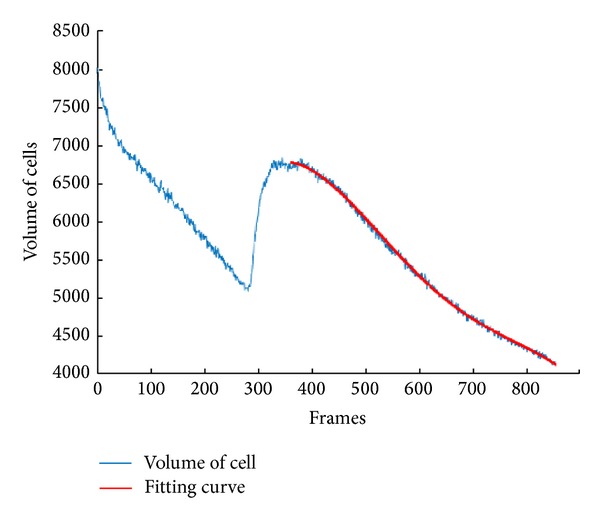
Fitting an exponential curve over the volume of cell behavior.

Figure 5 illustrates the detection of a representative maximum detected that corresponds to a connected element in one dimension space. The center of the connected element represents the maxima location, such that it is estimated with the mean of the connected elements; that is, *c*({*x*_*i*_∣*x*_*i*_ ∈ *R*(*x*_*i*_, *x*_*j*_)}) = (1/*n*)∑_*i*=1_^*n*^ *x*_*i*_, such that *R*(*x*_*i*_, *x*_*j*_) is an equivalent relation of the connectivity criterion. The behavior of the dynamic of the calcium for each particular cell should be modeled as a polynomial decay time-decreasing function as follows:

(9) $$\begin{array}{c} y=\overset{n}{\underset{i=0}{\sum }}a_{i}x^{i}, \end{array}$$

where *α*_*i*_ are the polynomial parameters, where the data used is taken from the maxima to the end of the data. The parameter estimation is performed by least squares the follow expression $\left[\begin{array}{c} a_{1}\\ \vdots \\ a_{n} \end{array}\right]={\left(X^{T}X\right)}^{-1}X^{T}y$, such that $X=\left[\begin{array}{ccc} x^{0} & \cdots & x^{n} \end{array}\right]$, for **x** data vertical vector. The order of polynomial is estimated from (*X* ^*T*^ *X*)^−1^ expression as follows: for a higher order *n*, the SVD decomposition of matrix (*X* ^*T*^ *X*)^−1^ = *S*Σ*V* ^*T*^ is used. The rank of matrix is estimated when normalized information of eigenvalues represents 99.99% of information; this is ∑_*j*=1_^*n*′^ *σ*_*j*_/∑_*i*=1_^*n*^ *σ*_*i*_ > 0.9999, where each *σ*_*i*_ is taken from matrix $\Sigma =\left[\begin{array}{ccc} \sigma_{1} & \cdots & 0\\ \vdots & \ddots & \vdots \\ 0 & \cdots & \sigma_{n} \end{array}\right]$ and *n*′ represent the polynomial order for fitting. For illustration purposes in Figure 6, a fitting sample is showed. The exponential help to model and analyze the decrease of the intensity registered in each cell.

### 4.3. Error Model Fitting

The correct construction of model over data is defined by introducing two measures of error: BIAS error and RMSE error. The first one is a measure of error modeling. The second measure is a precision error modeling criterion. The bias error provides information about how the model fits real data. Negative bias error means that model is undermodeling the data; that is, the model is a function under real data. Consequently, positive bias error represents overmodeling. Values near to zero mean that the model catches the dynamic of real data. Formally, bias error is defined as Bias(*x*, x*) = ∑_*i*=0_^*n*^ *x* − x*, where *x* represent real data and x* estimated data. Note that when BIAS is equal to zero it does not mean that the model is correct. It means that the same proportions of measures are below and under for real data. Then to quantify the precision error the RMSE is used. This error is the average of absolute differences among real and modeling data. RMSE is defined as follows: RMSE(*x*, x*) = (1/*n*)∑_*i*=1_^*n*^(x* − *x*)^2^ where x* represent modeling function and *x* real data.

### 4.4. Enhancement of Data

Although the least squared method offers the optimal model, it depends on the data measurement having normal distribution. Then, by the nature of the model, it results hard in verifying that these measures have a normal distribution. As a consequence it is necessary to enhance the data in order to facilitate the convergence of the approach. For simplicity, it is assumed that any signal *V*_*n*_(*i*), resulted from the calculation of the volume of an *n* cell for an *i* time, is affected by additive noise with zero mean as follows:

(10) $$\begin{array}{c} V_{n}=V_{n}^{*}+N_{n}, \end{array}$$

where *V*_n_* is the signal free noise and *N*_*n*_ is the additive noise with mean zero. In fact *N*_*n*_ has zero mean; the true signal data *V*_n_* is located into min⁡{dom⁡(*N*_*n*_)} and max⁡⁡{dom⁡(*N*_*n*_)} values. However, given *N*_*n*_ is a random variable, it locally should not present a zero mean, making it difficult to estimate the *V*_n_* value. To figure it out, it is needed to analyze locally the information, inferring the trend, and make an estimation of the expected value. The proposal consists of exploiting certain properties of operators taken from morphology operators. The reconstruction operators are useful because they approximate a surface by iterating successively a marker, getting the other surface that has similar topological properties. The approximation does not keep the original level of detail of its shape, such that it depends on the form and properties of structural element used. It should be considered inconvenient, but, in practical terms, it is its major advantage in sense and it represents the main trend of the original data, eliminating variations less than the structural element (high frequencies) of the original signal and resulting in a new signal that under- or overmodels the original data.

**Figure 7:**
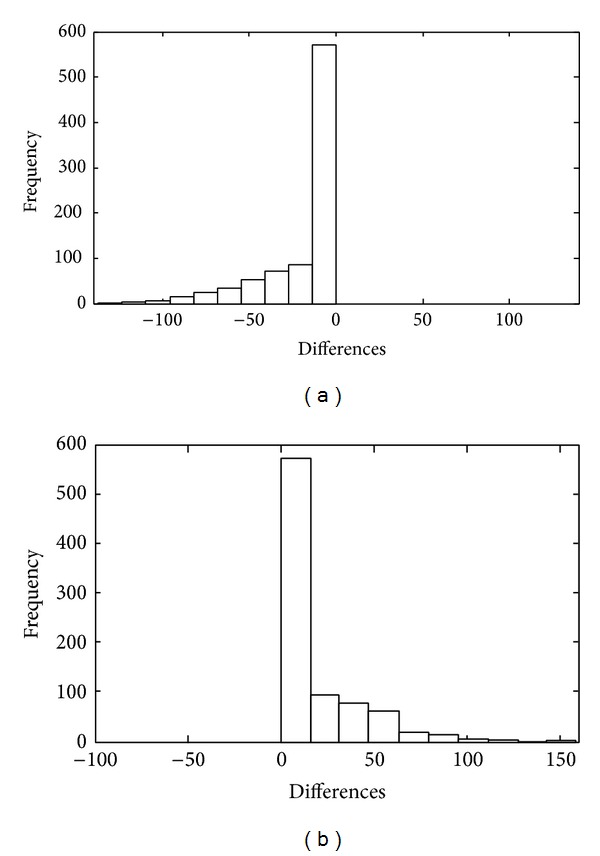
Histogram of differences from original signal and reconstructed signal (a) opening operator histogram, (b) closing operator histogram.

Considering the basic operators by reconstruction (opening and closing), the property of extensive or antiextensive, respectively, cause the fact that the application of each one over a signal *V*_*n*_ results in ${\tilde{\gamma }}_{\mu L}\left(V\right)$ or ${\tilde{\varphi }}_{\mu L}\left(V\right)$ signals such as under- or overmodeling the original. Both of them remain as the global trend of the topological information of *V*_*n*_. Consequently, the residual presents important topological information. However, the distribution of the data changes slightly: the shape of the derivative of the original and the approximated signal is different, changing the statistical properties of its PDFs.  Figure 7 presents the probability density function (PDF) approximated via its histogram after application over a signal *V*_*n*_. The histogram of opening operator presents a negative deviation, which means that the surface approximated is undermodeled. On the other hand, when we apply a closing operator, it overmodels the original signal and its histogram is deviated to the positive side of the range.

The proposal consists of mixing both filters, preserving the statistical information of the original signal. Noise effects are represented by the high frequencies. These frequencies must be discarded preserving the global trend of original signal *V*_*n*_. The discarded frequencies are directly related to the size of the structural element and the sampling process; that is, given a structural element of size *k*, it represents a temporality of *kf*, where *f* is the mean frequency of acquisition of *V*_*n*_. Then the process of filtering *f*(*V*_*n*_) is statistical consistent if and only if *V*_n_*, minus *V*_*n*_ preserve the following equality:

(11) $$\begin{array}{c} \;\rho \left(V_{n}-V_{n}^{*}\right)=G\left(0,\sigma \right). \end{array}$$

This is, the density distribution function of the difference between filtered data and original data is a normal distribution centered at the origin. The development of correct statistical filter must satisfy (9), where it is appreciated that opening and closing reconstruction operators provide negative and positive bias information of the approximated surface. The original signal is enveloped by the opening reconstruction and closing reconstruction, respectively, such that ${\tilde{\gamma }}_{\mu L}\left(V\right)\leq V_{n}\leq {\tilde{\varphi }}_{\mu L}\left(V\right)$. Consequently, for estimating *V*_*n*_, using ${\tilde{\gamma }}_{\mu L}\left(V\right)$ and ${\tilde{\varphi }}_{\mu L}\left(V\right)$ and considering that *E*[{*N*_1…*n*_}] = 0, and approximation to *V*_*n*_ is

(12) $$\begin{array}{c} f_{\mu }^{\mu L}\left(V\right)={\alpha_{1}\tilde{\gamma }}_{\mu L}\left(V\right)+\alpha_{2}{\tilde{\varphi }}_{\mu L}\left(V\right), \end{array}$$

where *α*_1_ and *α*_2_ are values between [0, 1] and its sum is the unit. In case that ${\tilde{\gamma }}_{\mu L}\left(V\right)$ and ${\tilde{\varphi }}_{\mu L}\left(V\right)$ use the same structural element, *α*_1_ = *α*_2_ = 0.5. In other cases these values would vary depending on effects of the geometry in the reconstruction process. The filter described above is denoted as a medium reconstruction filter. An extension of this filter implies a sequential form, where the properties of the structural element used in reconstruction stage should be varied as follows: let *p*(*μL*, *k*) be a function that returns a structural element with particular properties for *k* instant; sequential version of medium reconstruction filter is defined as

(13) $$\begin{array}{c} f_{\mu }^{p(\mu L,k)}\left(V\right)=f_{\mu }^{p(\mu L,k)}\cdot f_{\mu }^{p(\mu L,k-1)}\left(V\right)\cdot \cdots \cdot f_{\mu }^{p(\mu L,1)}\left(V\right). \end{array}$$

**Figure 8:**
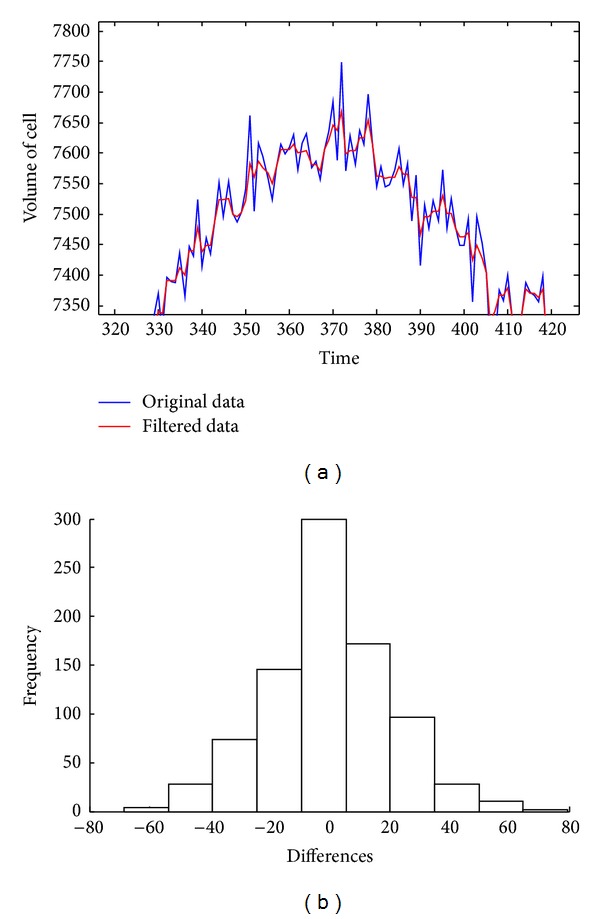
(a) Matching of original data and filtered data with the morphological medium filter. (b) Histogram of differences from original surface and reconstructed surface, as noted, the expected value is centered in zero and would be considered as a normal distribution.

Note that function *p*(*μL*, *k*) would vary the size and the topology of the structural element. The topology and size will affect the model that fits the data. The effect of applying the medium filter by reconstruction is illustrated in Figure 8, where in Figure 8(a) are presented the original data (blue color) and the filtered data (red color). As is appreciated, filtered signal follows the main trend of the original signal, discarding the high frequencies, and always statistical properties are kept as it is appreciated in Figure 8(b). This figure shows the difference of filtered image and original. This property makes it ideal for filtering data, improving the results when raw data fit the exponential decreasing function. For a detailed analysis the bias and RMSE error are showed for the case of filter and nonfilter signal. Bias error behaves to close in both scenarios; but the RMSE is deeply reduced, which means that the fitting process results are better, after filter data (Table 1).

## 5. Results and Discuss

**Figure 9:**
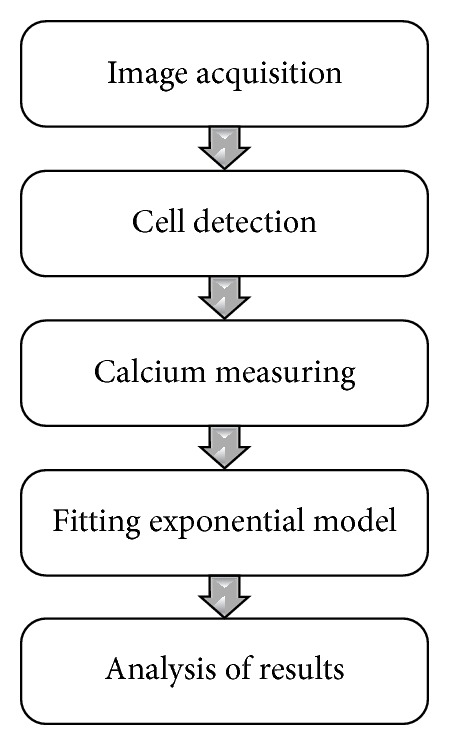
Block's diagram of the proposal.

**Figure 10:**
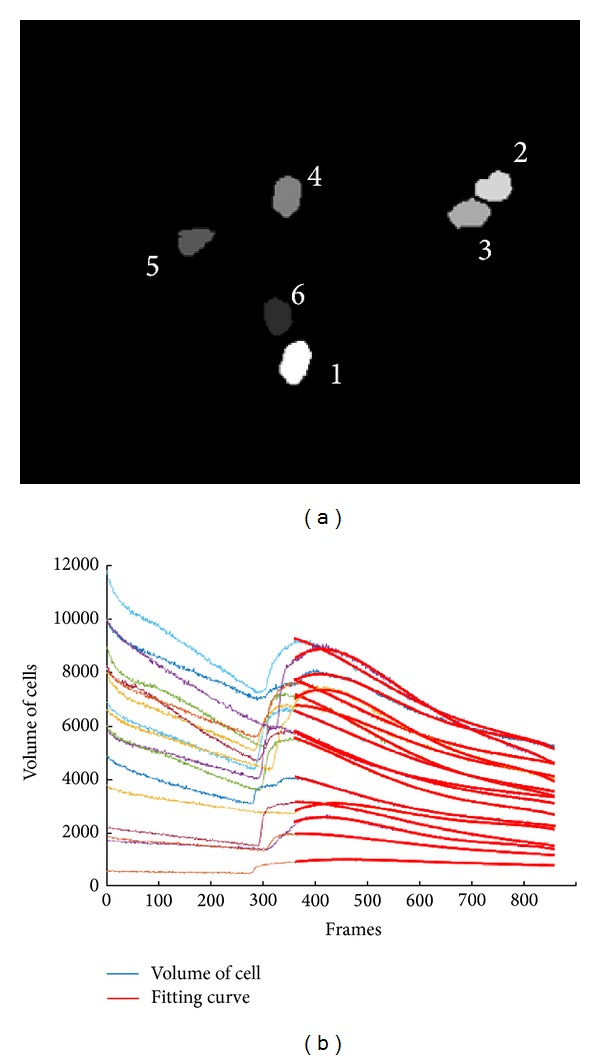
(a) Cells segmented, (b) curves calculated by the least squares fitting.

The proposal described above is tested under an experimental method that consists of analyzing a sequence of images that contains cells, which are exited applying Flour 4, in order to measure the effect over Ca^2+^ belonging to each cell and characterizing its behavior. The process is illustrated in Figure 9. The process diagram sums up the sequence of processing steps done over the sequence. The sequence of images was acquired from biological researchers of the Institute of Neurobiology, Campus UNAM-UAQ. The sequence was obtained from cells of* Xenopus laevis* frog. The calcium is measured indirectly with its excitation via Fluo-4 (by Molecular Probes). The optical material consists of a microscopy of fluorescence, setting up in an Olympus camera sensor IX71 at 485 to 520 wavelength sensitivity nm (excitation-emission, respectably); finally the images were acquired with fast acquisition camera (Evolution QEi Media Cybernetics), at 30 frames per second (Fps) with a resolution of 320 × 240 pixels. Finally, for testing purposes, 1,000 images have been selected, which represents temporary a sequence of 33-second length. The cell detection is a tough task because there are many factors which inside directly in the analysis process, as the nonhomogeneity luminance conditions of the images and the conditions that present the cells of interest. After the image acquisition, the following task consists of finding out and segmenting each cell. This process is performed via watershed approach. However, starting from the first frame acquired does not warrant correct cell detection. To make more robust the cell process detection, for each image, the cells are detected, as described in the third th section. Once the cells are detected, the neighborhood around the cell is considered to analyze the calcium concentration. The calcium concentration is performed by the measure of the luminance of each cell. The relation between the luminance intensity of the cell is highly correlated with the calcium concentration; that is, cells with high luminance have major calcium concentration. Next, the creation of the model of behavior results in a difficult task, because the behavior observed is not linear with the use of autoregression methods being inappropriate [15]. Then, discarding the times where the cell started to become excited, the dynamic of decreasing is modeled with an exponential function via least square method. The selected data includes the maxima location to the vanishing exited behavior. As recreated better the model, before applying least squared, the median filter by reconstruction is applied, which improve the accuracy of the modeling. Finally the results are showed in Figure 10. Observe, in Figure 10(a), that the cells are detected and the dynamic modeled as exponential superposed over measured data is showed (Figure 10(b)). The use of filter dismisses the high frequencies smoothing the behavior of luminance variations. The dismissing of high frequencies adds extra accuracy warranting that the exponential fitting has more significance, although the data are affected by noise effects. Finally, the way of how the cells were segmented represents a framework to analyze the intracellular calcium, which segment automatically the set of cells. This process is convenient in the sense that many of microscopic dynamics could be analyzed efficiently providing better information to the biologists.

## 6. Conclusions

In this paper, an automatic method for the study of intracellular calcium based on a marked controlled watershed transform for segmenting stage is presented. A new filter based on reconstruction operators is introduced. Then, having a high precision of cell segmenting and efficient ways to discard the noise measurement result the base for an automatic frameworks analysis as the experimentation shows. Finally, the reconstruction operators applied over one dimension data results usefully in the development of filters that help to create models of the dynamic of the calcium.

## Figures and Tables

**Table 1:** Errors of modeling with/without use of a median reconstruction filter.

Cell	Filtering		Without filter	
	BIAS	RMSE	BIAS	RMSE
1	0.0996	0.0415	0.0967	385.1000
2	0.5179	0.4742	0.1611	1304.4000
3	0.6913	1.7530	0.3772	7913.6000
4	1.1718	1.2087	0.3229	2906.9000
5	0.6538	0.7799	0.2144	2339.9000
6	0.7412	1.0012	0.1904	2306.6000
7	1.3467	1.2601	0.2079	1718.6000
8	1.1380	1.3794	0.2082	2282.1000
9	0.5010	0.4614	0.1823	1566.5000
10	1.4760	1.1212	0.1832	1198.6000
11	1.4346	0.9506	0.1091	620.1000
12	1.1730	1.6871	0.3167	4.1090
13	0.3320	0.2412	0.1132	0.5596
14	0.3510	0.1794	0.1601	703.2000
15	0.7043	0.7558	0.2293	2077.9000
16	1.6021	3.0539	0.2142	2721.2000
17	0.2594	0.1767	0.1605	999.4000
18	0.1688	0.0566	0.1144	343.6000
